# Exogenous tetracosahexaenoic acid modifies the fatty acid composition of human primary T lymphocytes and Jurkat T cell leukemia cells contingent on cell type

**DOI:** 10.1002/lipd.12372

**Published:** 2023-05-13

**Authors:** Nicola A. Irvine, Annette L. West, Johanna Von Gerichten, Elizabeth A. Miles, Karen A. Lillycrop, Philip C. Calder, Barbara A. Fielding, Graham C. Burdge

**Affiliations:** ^1^ School of Human Development and Health, Faculty of Medicine University of Southampton Southampton Hampshire UK; ^2^ Department of Nutritional Sciences, Faculty of Health and Medical Sciences University of Surrey Guildford Surrey UK; ^3^ Centre for Biological Sciences, Faculty of Natural and Environmental Sciences University of Southampton Southampton Hampshire UK; ^4^ National Institute of Health and Care Research Southampton Biomedical Research Centre University Hospital Southampton National Health Service Foundation Trust and University of Southampton Southampton Hampshire UK

**Keywords:** desaturases, docosahexaenoic acid, fatty acid metabolism, general area, immunology, lipid biochemistry, mammalian lipid biochemistry, metabolism, n‐3 fatty acids, polyunsaturated fatty acids (PUFA), specific lipids

## Abstract

Tetracosahexaenoic acid (24:6ω‐3) is an intermediate in the conversion of 18:3ω‐3 to 22:6ω‐3 in mammals. There is limited information about whether cells can assimilate and metabolize exogenous 24:6ω‐3. This study compared the effect of incubation with 24:6ω‐3 on the fatty acid composition of two related cell types, primary CD3^+^ T lymphocytes and Jurkat T cell leukemia, which differ in the integrity of the polyunsaturated fatty acid (PUFA) biosynthesis pathway. 24:6ω‐3 was only detected in either cell type when cells were incubated with 24:6ω‐3. Incubation with 24:6ω‐3 induced similar increments in the amount of 22:6ω‐3 in both cell types and modified the homeoviscous adaptations fatty acid composition induced by activation of T lymphocytes. The effect of incubation with 18:3ω‐3 compared to 24:6ω‐3 on the increment in 22:6ω‐3 was tested in Jurkat cells because primary T cells cannot convert 18:3ω‐3 to 22:6ω‐3. The increment in the 22:6ω‐3 content of Jurkat cells incubated with 24:6ω‐3 was 19.5‐fold greater than that of cells incubated with 18:3ω‐3. Acyl‐coA oxidase siRNA knockdown decreased the amount of 22:6ω‐3 and increased the amount of 24:6ω‐3 in Jurkat cells. These findings show exogenous 24:6ω‐3 can be incorporated into primary human T lymphocytes and Jurkat cells and induces changes in fatty acid composition consistent with its conversion to 22:6ω‐3 via a mechanism involving peroxisomal β‐oxidation that is regulated independently from the integrity of the upstream PUFA synthesis pathway. One further implication is that consuming 24:6ω‐3 may be an effective alternative means of achieving health benefits attributed to 20:5ω‐3 and 22:6ω‐3.

## INTRODUCTION

**FIGURE 1:**
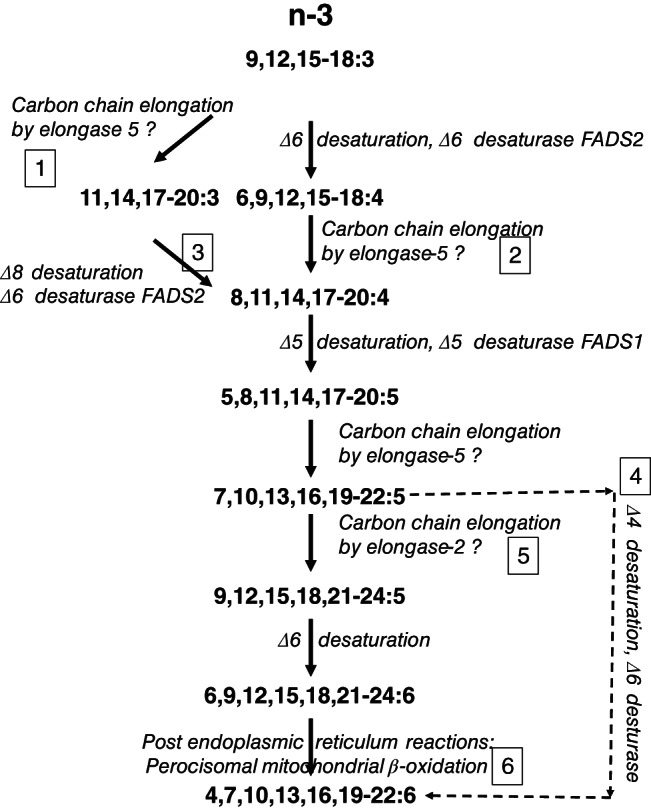
The ω‐3 polyunsaturated fatty acid biosynthesis pathway described in rat liver (Sprecher, 1999) plus the modifications found in human primary T lymphocytes (Robichaud et al., 2018; von Gerichten et al., 2021). (1) The first reaction in T lymphocytes is carbon chain elongation putatively by elongase‐5 (Sibbons et al., 2018; Robichaud et al., 2018; von Gerichten et al., 2021). (2) The first reaction in the hepatic pathway is Δ6 desaturation by the protein product of the *FADS2* gene followed by chain elongation by elongase‐5. (3) The protein product of the *FADS2* gene has Δ6 and Δ8 activities (Park et al., 2009), which are both expressed in Jurkat cells, while the Δ8 desaturase activity is predominant in T lymphocytes (Sibbons et al., 2018). (4) Desaturation at the Δ4 position is an alternative mechanism for 22:5ω‐3 synthesis in some cells 2:5ω‐3 synthesis in so (5) Elongase‐2 is not expressed in T lymphocytes (Robichaud et al., 2018; Sibbons et al., 2018; von Gerichten et al., 2021), therefore, truncating the pathway after synthesis of 22:5ω‐3. However, elongase‐2 is expressed in Jurkat cells (Sibbons et al., 2018). (6) The findings of (Moore et al., 1995; Voss et al., 1991) summarized by (Sprecher, 2000) suggest that the conversion of 24:6ω‐3 formed in the endoplasmic reticulum to 22:6ω‐3 involves translocation to peroxisomes and carbon chain shortening by one cycle of β‐oxidation.

Synthesis of longer‐chain ω‐3 polyunsaturated fatty acids (PUFAs) from the essential fatty acid α‐linolenic acid (18:3ω‐3) involves a pathway of mostly alternating desaturation and carbon chain elongation reactions that occur in the endoplasmic reticulum (Sprecher, 2000). In rodent (Voss et al., 1991) and human (Sibbons et al., 2014) hepatocytes, the first, rate‐limiting reaction is desaturation at the Δ6 position of 18:3ω‐3, which is catalyzed by the protein product of the *FADS2* gene, namely Δ6 desaturase, followed by the addition of two carbon atoms by elongase‐5 activity (Figure 1). Desaturation at the Δ5 position by Δ5 desaturase, yields 20:5ω‐3 which is converted by two cycles of chain elongation by elongase‐5 then elongase‐2 or ‐5 activities to form the intermediate 24:5ω‐3 which is converted to 24:6ω‐3 by Δ6 desaturase. Synthesis of 22:6ω‐3 involves translocation of 24:6ω‐3 from the endoplasmic reticulum to peroxisomes and removal of two carbon atoms by one cycle of β‐oxidation (Moore et al., 1995; Voss et al., 1991; Figure 1). The reactions downstream of 22:5ω‐3 synthesis has been suggested to regulate 22:6ω‐3 synthesis independently from the initial desaturation/elongation reactions (Burdge, 2004; Sprecher, 1999). This view is supported by the findings of whole body tracer studies using stable isotope labeled 18:3ω‐3, which showed sexual dimorphism in 22:6ω‐3 synthesis (Burdge et al., 2002; Burdge & Wootton, 2002; Pawlosky et al., 2003). Moreover, competition between exogenous 18:3ω‐3 and endogenous 24:5ω‐3 for Δ6 desaturase activity could modify the synthesis of the terminal product 22:6ω‐3 by reducing the desaturation of 24:5ω‐3 to 24:6ω‐3 (Burdge, 2022) which may explain the reduction in blood or tissue 22:6ω‐3 content in, at least, some 18:3ω‐3 dietary supplementation trials (Burdge, 2022; Gibson et al., 2013). Alternatively, direct synthesis of 22:6ω‐3 by desaturation at the Δ4 position of 22:5ω‐3 has been detected in MCF7 breast cancer cells that lack Δ6 desaturase activity (Grammatikos et al., 1994; Park et al., 2015) and a carnitine‐dependent mechanism for this reaction has also been proposed (Infante & Huszagh, 2000). One interpretation of these findings is that different cell types differ in their metabolic strategy for 22:6ω‐3 synthesis. Although 24:6ω‐3 is regarded as a metabolic intermediate that does not accumulate in tissues, rodents can convert dietary (Gotoh et al., 2018) or infused (Metherel et al., 2019) 24:6ω‐3 into 22:6ω‐3 in vivo and human skin fibroblasts can synthesize 22:6ω‐3 from radiolabeled 24:6ω‐3 in vitro (Moore et al., 1995), which suggest that at least some cell types can use exogenous 24:6ω‐3 as a substrate induction of proliferation of T lymphocytes involves homeoviscous adaptations in membrane fatty acid composition (Anel et al., 1990; Calder et al., 1994; Shires et al., 1989; von Gerichten et al., 2021) and in the relative proportions of phospholipid classes and individual molecular species (Ferber et al., 1975; Lonnberg et al., 2013) that are disrupted by incubation with ω‐3 PUFAs (Calder et al., 1994). Therefore, we investigated the effect of incubating purified quiescent or mitogen‐stimulated purified human CD3^+^ T lymphocytes with 24:6ω‐3 on their fatty acid composition as a proxy measure of 24:6ω‐3 conversion to 22:6ω‐3.

The widely studied Jurkat T lymphocyte model cell line (Abraham & Weiss, 2004) expresses *ELOVL2* and can synthesize 22:6ω‐3 from 18:3ω‐3 (Sibbons et al., 2018). We used Jurkat cells to compare the effects of exogenous 18:3ω‐3 and 24:6ω‐3 on fatty acid composition as a proxy measure to assess the relative effectiveness of 18:3ω‐3 and 24:6ω‐3 as substrates for 22:6ω‐3 synthesis. We also investigated whether peroxisomal and mitochondrial fatty acid β‐oxidation are involved in any 24:6ω‐3‐induced changes in Jurkat fatty acid composition.

## MATERIALS AND METHODS

### Ethics statement

The study was reviewed and approved by the East of England—Cambridge Central Research Ethics Committee (approval number 19/EE/0096) and all participants gave written informed consent. The purchase and use of primary T cells from StemCell Technologies UK Ltd. was reviewed and approved by the University of Southampton Faculty of Medicine Ethics Review Committee (submission I.D. 49658 and 58050.A1).

### Participants and collection of blood samples

The inclusion and exclusion criteria used to select participants in the study were described previously (von Gerichten et al., 2021). Briefly, donors were healthy men and women with an age of 41 (range 21–48) years (*n* = 10 [4 women]) and median body mass index 25.6 (24.1–26.5) kg/m^2^, blood pressure within age‐adjusted normal ranges, nonfasting total cholesterol concentration <7.5 mmol/L, HbA1c concentration <42 mmol/mol, and C‐reactive protein concentration <3 mg/L. Participants did not habitually consume fish oil, dietary oil supplements, smoke tobacco, or report any chronic disease. Volunteers were excluded if they did not meet the inclusion criteria, were pregnant or intending to become pregnant during the study, or were already participating in a clinical study. Nonfasting venous blood samples (100 mL) were collected into tubes containing lithium heparin.

### Isolation and culture of CD3 ^+^ T cells from whole blood

Peripheral blood mononuclear cells (PBMCs) were prepared from whole blood using a histopaque density cushion and centrifugation at 845*g* for 15 min at room temperature (von Gerichten et al., 2021). PBMCs were collected into RPMI1640 medium containing 10% (vol/vol) heat‐inactivated homologous pooled serum (Sigma‐Aldrich, Poole, UK; Complete medium; Table 1). CD3^+^ T cells were isolated by negative selection using the T cell EasySep kit (StemCell Technologies, UK Ltd., Cambridge, UK) according to the manufacturer's instructions. Isolated T cells were washed with 10 mL RPMI1640 and collected by centrifugation at 300*g* for 10 min at room temperature. CD3^+^ T lymphocytes were cryopreserved as described (Noakes et al., 2012; Prescott et al., 1999) and stored in liquid nitrogen. Blood donations by participants were suspended during the United Kingdom national restrictions to mitigate the SARS‐CoV‐2 pandemic. Consequently, to increase the sample number, purified CD3^+^ T lymphocytes were purchased from StemCell Technologies UK Ltd. (Cambridge, UK; Catalog number 70024.1); these cells were collected from anonymous donors whose characteristics met the inclusion criteria for the study.

**TABLE 1:** Fatty acid compositions of cell culture media.

	Proportions of fatty acids in culture media (moles %)		
Fatty acid	Culture medium	Culture medium plus 24:6ω‐3	Culture medium plus 18:3ω‐3
14:0	1.3	0.9	1.2
16:0	27.6	25.4	25.8
16:1ω‐7	2.2	1.9	2.0
18:0	8.8	8.7	8.6
18:1 ω‐9	19.9	19.1	18.7
18:1 ω‐7	1.4	1.5	1.4
18:2 ω‐6	30.3	28.0	28.2
18:3ω‐6	0.4	0.3	0.4
18:3ω‐3	0.5	0.4	6.6
20:0	<0.1	<0.1	0.1
20:1ω‐9	0.2	0.1	0.1
20:2ω‐6	0.2	0.2	0.2
20:3ω‐6	1.3	1.1	1.2
20:4ω‐6	4.8	4.3	4.6
20:5ω‐3	0.2	0.2	0.3
22:5ω‐3	0.4	0.2	0.2
22:6ω‐3	0.5	0.4	0.5
24:6ω‐3	ND	7.1	ND

*Note*: The total fatty acid composition of culture media was determined by gas chromatography as described in the Section 2. 24:6ω‐3 was not detected (ND) in media that were not supplemented with this fatty acid. Culture medium: RPMI1640 medium containing 10% (vol/vol) heat‐inactivated homologous pooled serum.

T lymphocyte culture was carried out as described (von Gerichten et al., 2021). Cryopreserved cells were thawed, adjusted to a density of 1 × 10^6^ cells/mL and incubated in RPMI1640 containing 2 mM l‐glutamine, 100 units/mL penicillin and 100 μg/mL streptomycin and 10% (vol/vol) heat‐inactivated pooled human serum (Sigma‐Aldrich) for 96 h with or without concanavalin A (10 μg/mL; Con. A; Sigma‐Aldrich), and either with or without 6(z), 9(z), 12(z), 15(z), 18(z), 21(z)‐24:6ω‐3 (30 μM; Cambridge Bioscience, UK) in a humidified incubator at 37°C in an atmosphere containing 5% (vol/vol) CO_2_. Jurkat cells were obtained from local stocks and maintained under the same conditions as T cells, without Con. A (Sibbons et al., 2018) for 96 h either with or without 24:6ω‐3 (30 μM; Cambridge Bioscience, UK) or 18:3ω‐3 (39 μM; Sigma Aldrich). Jurkat and T cells were collected by centrifugation, washed with unsupplemented RPMI1640 as before, and then snap‐frozen and stored at −80°C for fatty acid analysis. The purity of both 18:3ω‐3 and 24:6ω‐3 was greater than 95%.

In some experiments, Jurkat cells were treated with the carnitine palmitoyl transferase‐1 inhibitor Etomoxir (5 μM; Sigma‐Aldrich) together with 24:6ω‐3 (30 μM) for 48 h. Cells were collected by centrifugation and washed and processed for fatty acid analysis as before.

### siRNA knockdown of acyl‐CoA oxidase‐1 in Jurkat cells and RTPCR analysis

Jurkat cells were suspended in serum‐free Accell siRNA delivery media (Horizon Discovery Biosciences Ltd., Cambridge, UK) containing glutamine at the density of 1 × 10^6^ cells/mL and treated with either Accell human *ACOX1* SMARTPool siRNA (1 μM; Horizon Discovery Biosciences Ltd.) or nontargeted human pool siRNA (1 μM; Horizon Discovery Biosciences Ltd.) and incubated for 72 h at 37°C, in an atmosphere containing 5% (vol/vol) CO_2_. After 72 h, the plates were centrifuged at 300*g* for 10 min, the supernatant was removed and replaced with RPMI containing 10% human serum and 30 μM of 24:6n‐3. The cells were then incubated for a further 96 h. At the end of the incubation, the cells were collected, washed twice in PBS and pelleted for fatty acid composition analysis and to verify *ACOX1* knockdown. RNA extraction and qRTPCR were carried out essentially as described (von Gerichten et al., 2021). Briefly, RNA was extracted using the RNeasy Mini kit (Qiagen) with on‐column DNAse activity. RNA was eluted in RNase‐free water (30 μL). RNA concentration was measured and purity was assessed using a NanoDrop1000 spectrophotometer. cDNA was synthesized by reverse transcription. The level of the acyl‐CoA oxidase‐1 (ACOX1) transcript was measured by qRTPCR using QuantiTect assay Hs_ACOX1_1_SG (QT00078960) (Qiagen) with QuantiTect Sybr Green PCR kit (Qiagen). Amplified transcripts were quantified using the standard curve method (Cikos et al., 2007) and normalized to the geometric mean of the reference genes 60S ribosomal protein L13‐A (RPL13A, Quantitect Primer Assay Hs_RPL13A Primer design reference gene assay [HK‐SY‐hu]) and succinate dehydrogenase complex, subunit A, flavoprotein variant (SDHA), Quantitect Primer Assay Hs_SDHA_1_SG (QT00059486). These reference genes have been shown to be stable in CD3^+^ T lymphocytes and Jurkat cells (Sibbons et al., 2018) by the GeNorm method (Vandesompele et al., 2002). The qRTPCR conditions were those specified by the manufacturer.

### Analysis of fatty acid composition by gas chromatography

**FIGURE 2:**
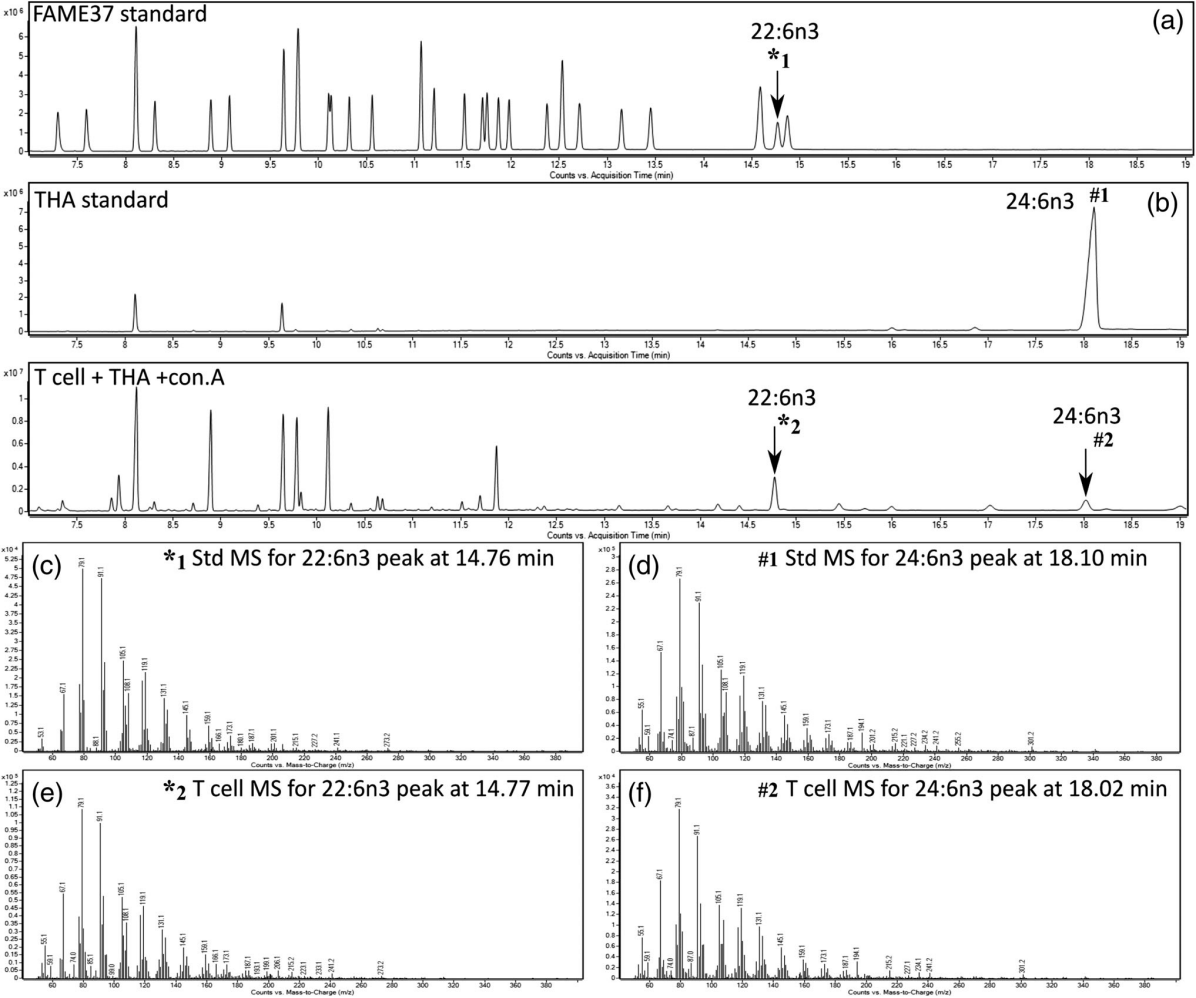
Confirmation of 24:6ω‐3 and 22:6ω‐3 peak identities by gas chromatography (GC)‐mass spectrometry (MS). (a) Separation of 37 FAMEs standard mixture by GC–MS, indicating the retention time of 22:6ω‐3 methyl ester peak (*1). (b) The retention time of 24:6ω‐3 methyl ester (#2) standard detected by GC–MS. (c) Mass spectrum of the 22:6ω‐3 methyl ester standard peak (*1), (d) mass spectrum of the 24:6ω‐3 (THA) methyl ester standard peak (#1), (e) mass spectrum of the 22:6ω‐3 methyl ester peak (*2) from CD3^+^ T lymphocytes. (f) Mass spectrum of the putative 24:6ω‐3 methyl ester peak (#2) from CD3^+^ T lymphocytes.

CD3^+^ T lymphocytes and Jurkat cells were thawed and suspended in 0.9% (wt/vol) NaCl and the internal standard 17:0 (3 μg) was added. Cell lipids were extracted with chloroform/methanol (2:1, vol/vol; Bligh & Dyer, 1959), dried, dissolved in toluene and converted to fatty acid methyl esters (FAMEs) by incubation with acidified methanol containing 2% (vol/vol) sulfuric acid at 50°C for 2 h (Burdge et al., 2000). The reaction mixture was cooled to room temperature and neutralized with KHCO_3_ (0.25 M) and K_2_CO_3_ (0.5 M). FAMEs were collected by hexane extraction (Burdge et al., 2000). FAMEs were separated on a BPX‐70 fused silica capillary column (30 m × 0.25 mm × 25 μm; Trajan, Scientific Europe, Milton Keynes, UK) using an Agilent 6890 gas chromatograph (GC) equipped with flame ionization detection as described (West et al., 2016). Chromatograms were integrated manually by a single operator using OpenLAB CDS ChemStation software (version BC.0301.001; Agilent Technologies, UK). The amounts of individual fatty acids are expressed as mass per million cells at the end of the culture period. Fatty acids were identified by their retention times relative to standards (37 FAMES, Sigma‐Aldrich) and confirmed by GC–mass spectrometry (Figure 2) using a 6890 gas chromatograph (Agilent, UK) equipped with an Agilent 5975 mass selective detector set to a mass scan range of *m*/*z* 50–550 as described (von Gerichten et al., 2021).

### Statistics

Statistical analyses were carried out using IBM SPSS Statistics for Windows, Version 27.0 (Armonk, NY: IBM Corp). The data were assessed for normality using the Kolmogorov Smirnov test. Normally distributed data are shown as mean ± SEM, while data that did not follow a normal distribution are reported as median (range). Pairwise comparisons for normally distributed data were made using Student's *t* test, or the Mann–Whitney *U* test for data that were not normally distributed. Statistical testing of the interaction between age and T cell activation status (activation state × life stage) was by 2‐way ANOVA with Tukey's post hoc correction for multiple comparisons. A sample size of *n* = 6 cultures or participants was calculated to provide 87% power to detect a significant difference in the amount of 22:6ω‐3 between treatments of 0.4 nmol/10^6^ cells with *α* <0.05. Effect sizes are reported either as Cohen's *d*, or partial eta squared.

## RESULTS

### The effect of incubation with 24:6ω‐3 on the fatty acid composition of T lymphocytes

There was a significant treatment × activation interaction effect on the amount of 24:0, but not on the amounts of any of the other SFAs or the MUFAs measured in T lymphocytes (Table 2).

**TABLE 2:** The effect of incubation with 24:6ω‐3 on activation‐induced changes in the fatty acid composition of CD3^+^ T lymphocytes.

	Amount of fatty acid (nmol/10^6^ cells)				ANOVA					
	Without 24:6ω‐3		With 24:6ω‐3		Tr	*ή* ^2p^	Ast	*p*	Tr x Ast	*ή* ^2^_p_
Fatty acid	Unstimulated	Stimulated	Unstimulated	Stimulated	*p*		*p*		*p*	
Saturated fatty acids										
14:0	0.32 ± 0.049^a^	0.39 ± 0.025	0.31 ± 0.028	0.44 ± 0.051	0.82	n.d.	0.01	n.d.	0.7	n.d.
16:0	0.97 ± 0.092	1.72 ± 0.183	0.76 ± 0.029	2.28 ± 0.139	0.14	n.d.	1.2e^−9^	0.44	0.3	n.d.
18:0	0.73 ± 0.072	0.97 ± 0.077	0.54 ± 0.028	1.10 ± 0.062	0.28	n.d.	1.2e^−14^	0.85	0.06	n.d.
20:0	0.01 ± 0.^001^	0.01 ± 0.000	0.00 ± 0.000	0.01 ± 0.001	0.55	n.d.	0.74	n.d.	0.4	n.d.
24:0	0.03 ± 0.003^a^	0.08 ± 0.008^b^	0.02 ± 0.001^a^	0.04 ± 0.004^a^	0.06	n.d.	0.07	n.d.	<0.001	0.39
Monounsaturated fatty acids										
16:1ω‐7	0.03 ± 0.004	0.09 ± 0.007	0.02 ± 0.002	0.11 ± 0.010	0.43	n.d.	7.5e^−14^	0.94	0.12	n.d.
18:1ω‐9	0.42 ± 0.040	1.06 ± 0.095	0.30 ± 0.013	1.23 ± 0.092	0.06	n.d.	0.02	0.23	0.36	n.d.
18:1ω‐7	0.08 ± 0.008^a^	0.14 ± 0.014	0.05 ± 0.^003a^	0.14 ± 0.008	0.07	n.d.	9.2e^−17^	0.97	0.3	n.d.
20:1ω‐9	0.02 ± 0.003^a^	0.04 ± 0.006	0.02 ± 0.003^a^	0.04 ± 0.002	0.12	n.d.	4.3e^−5^	0.57	0.53	n.d.
24:1ω‐9	0.03 ± 0.004^b^	0.02 ± 0.003	0.02 ± 0.001	0.02 ± 0.001	1.3e^−7^	0.76	0.6e^−5^	0.65	0.87	n.d.
ω‐6 Polyunsaturated fatty acids										
18:2ω‐6	0.56 ± 0.056^a^	1.32 ± 0.108^a^	0.42 ± 0.018^b^	1.52 ± 0.110^b^	0.0003	0.49	4.6e^−7^	0.73	1e^−5^	0.53
20:2ω‐6	0.01 ± 0.002	0.06 ± 0.010	0.01 ± 0.004	0.06 ± 0.005	0.49	n.d.	0.69	n.d	0.22	n.d
20:3ω‐6	0.05 ± 0.004	0.15 ± 0.020	0.04 ± 0.002	0.12 ± 0.012	0.86	n.d	1.1e^−5^	0.54	0.12	n.d
20:4ω‐6	0.44 ± 0.039^a^	0.57 ± 0.044^a^	0.32 ± 0.020	0.54 ± 0.028	0.001	0.42	4.3e^−5^	0.78	0.006	0.32
ω‐3 Polyunsaturated fatty acids										
18:3ω‐3	0.01 ± 0.002	0.01 ± 0.001	0.01 ± 0.004	0.02 ± 0.001	0.19	n.d.	0.60	n.d.	0.40	n.d.
20:3ω‐3	0.03 ± 0.003	0.10 ± 0.036	0.02 ± 0.002	0.03 ± 0.010	0.001	0.43	9.3e^−8^	0.47	0.33	n.d.
20:5ω‐3	0.01 ± 0.002	0.01 ± 0.001	0.01 ± 0.00^a^	0.02 ± 0.00^b^	0.85	n.d.	0.01	0.27	0.28	n.d.
22:5ω‐3	0.02 ± 0.002a	0.04 ± 0.004^b^	0.01 ± 0.001^a^	0.04 ± 0.003^b^	0.48	n.d.	5.7e^−8^	0.78	0.06	n.d.
22:6ω‐3	0.02 ± 0.003^a^	0.04 ± 0.006^b^	0.05 ± 0.004^b^	0.28 ± 0.038^c^	7.5e^−7^	0.55	0.01	0.31	0.04	0.19
24:6ω‐3	0.00 ± 0.000	0.00 ± 0.000^a^	0.00 ± 0.00^a^	0.13 ± 0.019^b^	3.3e^−12^	0.92	0.02	0.53	0.19	n.d.

*Note*: Values are mean ± SEM amounts of T lymphocyte fatty acids from six different participants per treatment. Statistical comparisons were done by two‐way ANOVA with treatment (Tr; incubation with or without 24:6ω‐3) and activation status (Ast) as fixed factors. Testing post hoc was performed by Tukey's method. *p* Values are reported for single factor and Tr × Ast interaction effects. Effect sizes of means that differed significantly (*p* < 0.05) are reported asp artial Eta squared *ή*_p_ but were not determined (n.d.) for comparisons that failed to reach statistical Means that do not share superscripted letters differ significantly.

Mitogen stimulation significantly increased the amounts of 18:2ω‐6 (2.4‐fold) and 20:4ω‐6 (1.3‐fold) and there was a significant treatment × activation interaction effect on the amount of 20:4ω‐6, but the amounts of the other ω‐6 PUFAs that were measured in T cells were not altered (Table 2). Specifically, the mitogen‐induced increment in 20:4ω‐6 was greater in T lymphocytes incubated with 24:6ω‐3 than in untreated cells. The activation‐induced change in the amount of 24:1ω‐9 was blunted in T cells incubated with 24:6ω‐3 compared to untreated T lymphocytes.

The identities of 24:6ω‐3 and 22:6ω‐3 were confirmed by comparison of the mass spectra of authentic standards with those of the peaks with the corresponding retention times in T lymphocytes (Figure 2). 24:6ω‐3 was not detected in quiescent or activated CD3^+^ T cells that were maintained in medium lacking this fatty acid (Table 2, Figure 2), but was significantly incorporated into 24:6ω‐3 treated T lymphocytes irrespective of activation status (Table 2). Incubation with 24:6ω‐3 increased the amount of 22:6ω‐3 in quiescent (fivefold) and activated (sevenfold) T cells compared to untreated cells (Table 2). Mitogen stimulation increased the amount of 20:3ω‐3 in T cells that were not incubated with 24:6ω‐3. However, the magnitude of mitogen‐induced change in 20:3ω‐3 content was less in cells incubated with 24:6ω‐3 than in untreated T cells. There was no single factor effect of 24:6ω‐3 on the amounts of 20:5ω‐3 or 22:5ω‐3, but there was a significant treatment × activation effect on the amounts of these ω‐3 PUFAs (Table 2).

### The effect of incubation with 24:6ω‐3 or 18:3ω‐3 on the fatty acid composition of Jurkat cells

Neither 24:6ω‐3 nor 18:3ω‐3 significantly altered the SFA or MUFA contents of Jurkat cells (Table 2). Incubation with 24:6ω‐3 significantly decreased the amounts of 20:3ω‐6 (30%) and 20:4ω‐6 (51%), but there was no significant effect of incubation with 24:6ω‐3 on the 18:2ω‐6 and 20:2ω‐6 contents of Jurkat cells (Table 3). In contrast, incubation with 24:6ω‐3 increased the amounts of 20:5ω‐3 (68%), 22:5ω‐3 (19%), 24:6ω‐3 (0–9 nmol/million cells) and 22:6ω‐3 (6.9‐fold) in Jurkat cells. Incubating Jurkat cells with 18:3ω‐3 significantly increased the amounts of 18:3ω‐3 (37‐fold), 20:3ω‐3 (16.6‐fold), 20:5ω‐3 (13‐fold), and 22:5ω‐3 (6.5‐fold), while the increment in 22:6ω‐3 (5%) did not reach statistical significance (Table 3). The increment in the amount of 22:6ω‐3 induced in Jurkat cells by incubation with 24:6ω‐3 was 19.5‐fold greater than the increase due to incubation with 18:3ω‐3. 20:4ω‐3 was not detected in Jurkat cells after adjustment for the final concentrations of these substrates.

**TABLE 3:** The effect of incubation with 24:6ω‐3 or 18:3ω‐3 on the fatty acid composition of Jurkat cells.

	Amount of fatty acid (nmol/10^6^ cells)		*t* Test		Amount of fatty acid (nmol/10^6^ cells		*t* Test	
Fatty acid	Without 24:6ω‐3	With 24:6ω‐3	*p*	Cohen's *d*	Without 18:3ω‐3	With 18:3ω3	*p*	Cohen's *d*
Saturated fatty acids								
14:0	2.15 ± 0.14	1.98 ± 0.10	0.93	n.d.	0.29 ± 0.04	0.28 ± 0.04	0.7	n.d.
16:0	22.76 ± 0.13	21.56 ± 0.33	0.62	n.d.	2.58 ± 0.33	2.67 ± 0.60	0.38	n.d.
18:0	13.30 ± 0.09	11.81 ± 0.20	0.73	n.d.	1.24 ± 0.15	1.34 ± 0.33	0.80	n.d.
20:0	0.19 ± 0.01	0.18 ± 0.01	0.46	n.d.	0.01 ± 0.00	0.01 ± 0.00	0.31	n.d.
24:0	5.10 ± 0.07	3.11 ± 0.18	0.13	n.d.	0.31 ± 0.03	0.33 ± 0.08	0.18	n.d.
Monounsaturated fatty acids								
16:1ω‐7	1.16 ± 0.01	1.13 ± 0.02	0.62	n.d.	0.15 ± 0.02	0.13 ± 0.03	0.72	n.d.
18:1ω‐9	20.81 ± 0.05	17.53 ± 0.08	0.76	n.d.	1.72 ± 0.21	2.00 ± 0.50	0.62	n.d.
18:1ω‐7	3.93 ± 0.02	2.90 ± 0.03	0.99	n.d.	0.29 ± 0.04	0.39 ± 0.10	0.38	n.d.
20:1ω‐9	1.90 ± 0.03	1.88 ± 0.02	0.99	n.d.	0.16 ± 0.02	0.21 ± 0.05	0.39	n.d.
24:1ω‐9	0.28 ± 0.01	0.32 ± 0.01	0.82	n.d.	0.15 ± 0.02	0.13 ± 0.01	0.10	n.d.
ω‐6 Polyunsaturated fatty acids								
18:2ω‐6	7.98 ± 0.06	9.28 ± 0.23	0.07	n.d.	1.04 ± 0.12	0.85 ± 0.21	0.45	n.d.
20:2ω‐6	1.46 ± 0.02	1.52 ± 0.02	0.036	0.19	0.16 ± 0.02	0.16 ± 0.04	0.95	n.d.
20:3ω‐6	6.82 ± 0.04	4.74 ± 0.19	0.08	n.d.	0.50 ± 0.06	0.57 ± 0.14	0.64	n.d
20:4ω‐6	9.36 ± 0.08	5.95 ± 0.29	0.001	1.65	0.41 ± 0.05	0.62 ± 0.1	0.22	n.d
ω‐3 Polyunsaturated fatty acids								
18:3ω‐3	0.05 ± 0.01	0.17 ± 0.01	0.72	n.d.	0.01 ± 0.01	0.37 ± 0.04	1.0e^−6^	4.59
20‐3ω‐3	0.60 ± 0.04	0.49 ± 0.15	0.07	1.16	0.03 ± 0.01	0.21 ± 0.03	0.005	3.77
20:5ω‐3	0.16 ± 0.01	0.27 ± 0.01	2e^−5^	0.56	0.01 ± 0.00	0.13 ± 0.01	5.1e^−8^	4.58
22:5ω‐3	1.03 ± 0.01	1.23 ± 0.04	1.6e^−5^	0.04	0.06 ± 0.01	0.39 ± 0.05	3.3e^−9^	4.07
22:6ω‐3	0.77 ± 0.01	5.32 ± 0.20	2.2e^−9^	0.15	0.57 ± 0.00	0.60 ± 0.00	0.53	n.d.
24:6ω‐3	0.00 ± 0.01	8.98 ± 0.21	3e^−5^	0.23	0.00 ± 0.01	0.00 ± 0.01	0.55	n.d.

*Note*: Values are mean ± SEM amounts of fatty acids (*n* = 6 culture replicates per treatment). Statistical comparisons were done by Student's unpaired *t* test (equal variances were not assumed). Effect sizes of means that differed significantly (*p* < 0.05) are reported as Cohen's *d*, but were not determined (n.d.) for comparisons that failed to meet the threshold for statistical significance.

### Effect of acyl‐CoA oxidase‐1 siRNA knockdown on the fatty acid composition of Jurkat cells incubated with 24:6ω‐3

**FIGURE 3:**
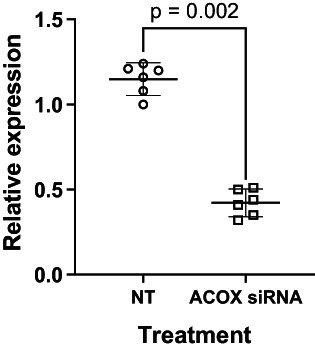
The effect of treatment with *ACOX1* siRNA on *ACOX1* mRNA expression in Jurkat cells. Values are median (95% confidence interval, *n* = 6 culture replicates/treatment) acyl‐coA oxidase‐1 (ACOX) mRNA levels from individual Jurkat cell cultures treated for 48 h with either ACOX siRNA or nontargeted siRNA (NT). Statistical comparison was by the Mann–Whitney *U* test.

Incubation of Jurkat cells with ACOX1 siRNA reduced the median level of the ACOX1 mRNA transcript by 65% (*p* = 0.0006; Mann Whitney *U*‐test) compared to cells incubated with the nontargeted control siRNA (Figure 3). ACOX1 siRNA knockdown in Jurkat cells incubated with 24:6ω‐3 increased the amount of 20:0 by 1.5‐fold compared to cells treated with NT siRNA, but there were no statistically significant differences in the amounts of any of the other SFAs measured (Table 4). Jurkat cells treated with ACOX1 siRNA plus 24:6ω‐3 contained 68% more 18:1ω‐7 than cells treated with NT siRNA, while there were no significant differences between treatments in the amounts of the other MUFAs measured (Table 4). Treatment of Jurkat cells with ACOX1 siRNA plus 24:6ω‐3 significantly increased the amount of 20:2ω‐6 (43%), 20:3ω‐6 and 24:6ω‐3 compared to cells treated with NT siRNA plus 24:6ω‐3 (Table 4). Jurkat cells incubated with ACOX1 siRNA plus 24:6ω‐3 contained 24% less 20:3ω‐3, 23% less 22:5ω‐3, and 36% less 22:6ω‐3, but twofold more 20:5ω‐3 than cells treated with NT siRNA plus 24:6ω‐3.

**TABLE 4:** The effect of acyl‐CoA oxidase‐1 SiRNA knockdown and Etomoxir treatments on the fatty acid composition of Jurkat cells incubated with 24:6ω‐3.

	Amount of fatty acid (nmol/10^6^ cells)		*t* Test		Amount of fatty acid (nmol/10^6^ cells)		*t* Test	
Fatty acid	NT siRNA +24:6ω‐3	*ACOX1* siRNA +24:6ω‐3	*p*	Cohen's *d*	Etomoxir control +24:6ω‐3	Etomoxir +24:6ω‐3	*p*	Cohen's *d*
Saturated fatty acids								
14:0	0.45 ± 0.06	0.41 ± 0.11	0.93	n.d.	1.01 ± 0.12	0.25 ± 0.05	0.03	n.d.
16:0	17.36 ± 2.87	20.82 ± 0.41	0.62	n.d.	21.60 ± 0.20	7.51 ± 0.35	0.19	n.d.
18:0	0.66 ± 0.10	0.76 ± 0.11	0.73	n.d.	13.75 ± 0.25	4.62 ± 0.15	0.06	n.d.
20:0	13.42 ± 2.58	20.35 ± 1.10	0.046	1.8	1.85 ± 0.03	0.08 ± 0.00	0.07	n.d.
Monounsaturated fatty acids								
16:1ω‐7	11.62 ± 1.78	19.51 ± 0.26	0.09	n.d.	0.98 ± 0.04	0.76 ± 0.11	0.44	n.d.
18:1ω‐9	2.67 ± 0.43	4.85 ± 0.08	0.76	n.d.	16.93 ± 0.24	19.51 ± 0.26	0.71	n.d.
18:1ω‐7	4.82 ± 0.68	8.09 ± 0.27	0.019	0.17	3.44 ± 0.02	4.85 ± 0.08	0.01	0.27
20:1ω‐9	0.32 ± 0.08	0.31 ± 0.08	0.99	n.d.	1.59 ± 0.03	1.74 ± 0.07	0.44	n.d
24:1ω‐9	0.18 ± 0.03	0.40 ± 0.02	0.34	n.d.	1.33 ± 0.02	0.55 ± 0.02	0.04	9.21
ω‐6 Polyunsaturated fatty acids								
18:2ω‐6	1.15 ± 0.14	1.74 ± 0.07	0.08	n.d.	8.02 ± 0.18	8.09 ± 0.27	0.82	n.d.
20:2ω‐6	0.91 ± 0.11	1.30 ± 0.05	0.04	0.39	0.17 ± 0.01	1.30 ± 0.05	0.85	n.d.
20:3ω‐6	2.35 ± 0.33	2.79 ± 0.09	0.01	0.89	3.97 ± 0.02	2.79 ± 0.09	0.23	n.d.
20:4ω‐6	3.00 ± 0.46	4.64 ± 0.10	0.001	0.65	4.95 ± 0.03	4.64 ± 0.10	0.01	4.42
ω‐3 Polyunsaturated fatty acids								
18:3ω‐3	0.14 ± 0.02	0.33 ± 0.01	0.54	n.d.	0.22 ± 0.01	0.41 ± 0.08	0.16	4.6
20:3ω‐3	0.33 ± 0.05	0.25 ± 0.02	0.07	n.d.	0.46 ± 0.01	0.33 ± 0.01	1.94	n.d.
20:5ω‐3	0.28 ± 0.04	0.55 ± 0.02	0.14	n.d.	2.05 ± 0.03	2.13 ± 0.07	0.29	n.d.
22:5ω‐3	0.95 ± 0.14	0.73 ± 0.02	0.56	n.d.	1.33 ± 0.02	0.73 ± 0.02	0.04	0.34
22:6ω‐3	4.91 ± 0.42	3.14 ± 0.17	0.01	0.23	10.22 ± 0.08	5.49 ± 0.17	0.01	0.76
24:6ω‐3	2.69 ± 0.25	3.27 ± 0.13	0.002	0.15	6.85 ± 0.34	4.27 ± 0.13	0.02	0.211

*Note*: Values are mean ± SEM (*n* = 6 culture replicates per treatment). All cultures contained 24:6ω‐3 (25 μM). Statistical comparisons were done by Student's unpaired *t* test (equal variances were not assumed). Effect sizes of means that differed significantly (*p* < 0.05) are reported as Cohen's *d*, but were not determined (n.d.) for comparisons which failed to meet the threshold for statistical significance.

Abbreviations: ACOX1, acyl‐CoA oxidase; NT non‐targeted siRNA control.

### The effect of treatment with Etomoxir on the fatty acid composition of Jurkat cells incubated with 24:6ω‐3

There was no significant effect of Etomoxir treatment on the amounts of SFAs, while the amount of 18:1ω‐7 was greater (1.4‐fold) in Jurkat cells treated with Etomoxir plus 24:6ω‐3 than Jurkat cells incubated with 24:6ω‐3 alone (Table 3). Treatment of Jurkat cells with Etomoxir plus 24:6ω‐3 reduced the amount of 20:4ω‐6 by 6%, while there was no significant effect on the amounts of the other ω‐6 PUFAs measured. Treatment of Jurkat cells with Etomoxir plus 24:6ω‐3 reduced the amounts of 22:6ω‐3, 24:6ω‐3, and 22:5ω‐3 by 46%, 38%, and 45%, respectively. There were no statistically significant effects of treatment with Etomoxir plus 24:6ω‐3 on the amounts of the other ω‐3 PUFAs measured (Table 3).

## DISCUSSION

Overall, these findings show that although 24:6ω‐3 has been regarded as a poor substrate for phospholipid biosynthesis (Voss et al., 1991), both primary CD3+ T lymphocytes and Jurkat cells accumulated 24:6ω‐3 when incubated with exogenous 24:6ω‐3, which was greater in activated T cells than unstimulated cells, which is consistent with the general increase in the uptake of exogenous fatty acids by mitogen‐stimulated T cells (Rode et al., 1982). Exogenous 24:6ω‐3 can be incorporated into primary human T lymphocytes and Jurkat cells The cell type‐related changes in fatty acid composition induced by treatment with 24:6 ω‐3 and acyl‐coA oxidase knockdown are which suggests conversion of 24:6ω‐3 to 22:6ω‐3 via a mechanism involving peroxisomal β‐oxidation that is regulated independently from the upstream reactions of the PUFA synthesis pathway (Figure 1). In addition, one possible explanation for increased amounts of saturated or monounsaturated fatty acids are that they represent homeoviscotic adaptations induced by the accumulation of 24:6 ω‐3. However, because of such homeoviscotic adaptations and uptake of fatty acids from the culture medium, the changes in T cell fatty acid composition induced by incubation with 24:6ω‐3 cannot be assumed to reflect metabolic interconversions alone. Nevertheless, the results of previous studies (Metherel et al., 2019; Metherel & Bazinet, 2019; Moore et al., 1995) support the interpretation of the present findings as showing that both CD3^+^ T lymphocytes and Jurkat cells can utilize 24:6ω‐3 as a substrate for 22:6ω‐3 synthesis and that such interconversion can occur irrespective of the integrity of the PUFA synthesis pathway. One interpretation of the similarity between Jurkat cells and primary T lymphocytes in the amount of 22:6ω‐3 following incubation with 24:6ω‐3 is that the post‐endoplasmic reticulum reactions of the PUFA synthesis pathway can be regulated independently from the preceding metabolic steps as suggested previously (Burdge, 2004; Sprecher, 1999).

Fibroblasts from patients with Zellweger's disease who lack peroxisomes do not synthesize 22:6ω‐3 which supports the conclusion that peroxisomal fatty acid β‐oxidation is required for 22:6ω‐3 synthesis (Moore et al., 1995). However, despite this metabolic block, accumulation of 24:6ω‐3 has not been reported in tissues or blood from patients who lack peroxisomes (Martinez, 1995) or from *Pex‐2*/*Pex‐5* dual knockout mice that do not synthesize peroxisomes or express enzymes involved in peroxisomal fatty acid β‐peroxidation (Baes et al., 1997; Faust & Hatten, 1997; Janssen et al., 2000). Moreover, *Pex*‐2/*Pex*‐5 null mice did not differ in liver 22:6ω‐3 content from peroxisome replete mice (Janssen et al., 2000). Therefore, the role of peroxisomal fatty acid β‐oxidation in 22:6ω‐3 biosynthesis remains a matter for debate (Infante & Huszagh, 2001). Direct synthesis of 22:5ω‐3 by Δ4 desaturation by the protein product of *FADS2* (Park et al., 2015) or by a carnitine plus α‐tocopherol‐dependent mitochondrial pathway (Infante & Huszagh, 2000) have been suggested as alternative mechanisms. Jurkat cells were treated with *ACOX1* siRNA in order to investigate whether peroxisomal fatty acid β‐oxidation was involved in, at least, some of the changes in fatty acid composition induced by incubation with 24:6ω‐3, The present findings show that 64% reduction in *ACOX1* mRNA expression by transfection of Jurkat cells with *ACOX1* siRNA was accompanied by lower amounts of 22:6ω‐3, 22:5ω‐3, 20:5ω‐3 and 20:3ω‐3, and more 24:6ω‐3 when cells were incubated with 24:6ω‐3 alone. This finding suggests that peroxisomal fatty acid β‐oxidation is involved in the synthesis of other ω‐3 PUFAs as well as 22:6ω‐3, at least in Jurkat leukemia cells, although this interpretation requires more rigorous testing by more direct methods. To the best of our knowledge, the mechanism by which 24:6ω‐3 could be converted to 20:5ω‐3 has not been described, although it is possible that this may occur via the recycling of carbon atoms from peroxisomal fatty acid β‐oxidation and utilized in the conversion of 18:3ω‐3 to 20:3ω‐3, although such recycling of carbon atoms from ω‐3 PUFAs has only been reported for labeled 18:3ω‐3 which can be utilized in cholesterol synthesis in rodent brain (Cunnane et al., 1994) and whole body SFA and MUFA synthesis in humans (Burdge & Wootton, 2003) and rhesus macaques (Sheaff Greiner et al., 1996). The present findings imply that the suggestion that conversion of 24:6ω‐3 is a minor source of ω‐3 PUFAs (Metherel & Bazinet, 2019) may depend on cell type.

Treatment of Jurkat cells with Etomoxir differentially decreased the amounts of 24:6ω‐3, 22:5ω‐3, 22:6ω‐3, 18:1ω‐7, and 20:4ω‐6 in the cells. One possible interpretation is that the synthesis of these unsaturated fatty acids involves mitochondrial β‐oxidation, for example by carbon recycling at least in Jurkat cells as occurs in T lymphocytes incubated with [^13^C‐18:3 ω‐3] (West et al., 2022). In the absence of findings from experiments using a 24:6ω‐3 tracer, it is not possible to draw robust conclusions about the mechanism of 24:6ω‐3 metabolism in T lymphocytes or Jurkat cells, which is an important limitation of the present study.

24:6ω‐3 typically accounts for less than 2% of the total fatty acids in marine fish (Tomita & Ando, 2009), but up to 13% in some species of starfish (Suo et al., 2015) and jellyfish (Nichols et al., 2003). Jellyfish are consumed frequently in Asian Countries, but rarely in Europe and North America (Bonaccorsi et al., 2020). The finding that human T cells can assimilate and metabolize 24:6ω‐3, possibly with greater efficiency than 18:3ω‐3, suggests that consuming foods that contain 24:6ω‐3 could be an alternative to oily fish for achieving the benefits associated with 20:5ω‐3 and 22:6ω‐3 (Calder, 1997, 1998).

## AUTHOR CONTRIBUTIONS

Graham C. Burdge, Barbara A. Fielding, Philip C. Calder, Elizabeth A. Miles, and Karen A. Lillycrop conceived and designed the study. Johanna Von Gerichten, Annette L. West, and Nicola A. Irvine conducted the experiments and analyzed the data with Graham C. Burdge. Graham C. Burdge wrote the first draft of the manuscript. All authors contributed to drafting the manuscript and approved the submitted version.

## ACKNOWLEDGMENTS

This work was supported by grants from the Biotechnology and Biological Sciences Research Council (BB/S00548X/1 and BB/S005358/1). Philip C. Calder and Graham C. Burdge are supported by the National Institute for Health and Care Research through the NIHR Southampton Biomedical Research Centre. Neither funder was involved in the study design, collection, analysis, interpretation of data, the writing of this article, or the decision to submit the manuscript for publication. Publication costs were paid by the University of Southampton.

## CONFLICT OF INTEREST STATEMENT

The authors declare that they have no conflict of interest.

## DECLARATIONS

Graham C. Burdge has received research funding from Nestle, Abbott Nutrition, and Danone and has served as a member of the Scientific Advisory Board of BASF. Philip C. Calder acts as a consultant to BASF, Smartfish, DSM, Cargill, Danone/Nutricia, and Fresenius‐Kabi. Karen A. Lillycrop has received research funding from Nestle, Abbott Nutrition, and Danone.
